# Efficient solar-driven electrocatalytic CO_2_ reduction in a redox-medium-assisted system

**DOI:** 10.1038/s41467-018-07380-x

**Published:** 2018-11-27

**Authors:** Yuhang Wang, Junlang Liu, Yifei Wang, Yonggang Wang, Gengfeng Zheng

**Affiliations:** Laboratory of Advanced Materials, Department of Chemistry and Shanghai Key Laboratory of Molecular Catalysis and Innovative Materials, Collaborative Innovation Center of Chemistry for Energy Materials, Fudan University, Shanghai, 200438 China

## Abstract

Solar-driven electrochemical carbon dioxide (CO_2_) reduction is capable of producing value-added chemicals and represents a potential route to alleviate carbon footprint in the global environment. However, the ever-changing sunlight illumination presents a substantial impediment of maintaining high electrocatalytic efficiency and stability for practical applications. Inspired by green plant photosynthesis with separate light reaction and (dark) carbon fixation steps, herein, we developed a redox-medium-assisted system that proceeds water oxidation with a nickel-iron hydroxide electrode under light illumination and stores the reduction energy using a zinc/zincate redox, which can be controllably released to spontaneously reduce CO_2_ into carbon monoxide (CO) with a gold nanocatalyst in dark condition. This redox-medium-assisted system enables a record-high solar-to-CO photoconversion efficiency of 15.6% under 1-sun intensity, and an outstanding electric energy efficiency of 63%. Furthermore, it allows a unique tuning capability of the solar-to-CO efficiency and selectivity by the current density applied during the carbon fixation.

## Abstract (web summary)

Generating high-energy fuels from sunlight, water, and CO_2_ using synthetic materials requires, among many things, the careful separation of reduced and oxidized products. Here, authors employ a zinc-based redox pair to spatially and temporally separate light-driven water oxidation and CO_2_ reduction.

## Introduction

The fast-growing atmospheric CO_2_ level is producing a significant environment crisis for the world today. The sunlight energy-driven electrochemical conversion of CO_2_ into various value-added chemicals^1^ such as carbon monoxide (CO)^2–4^, formic acid^5,6^, methane^7,8^, ethylene^9,10^ and ethanol^11^, known as the artificial photosynthesis, is regarded as a promising solution to alleviate this urgent environment pressure^12,13^, as well as to store the sunlight energy into chemical fuels or electricity. Compared to the other configurations of artificial photosynthetic systems such as photocatalysis^14^ and photoelectrochemical conversion^15^, electrocatalysis powered by photovoltaics has so far shown the highest solar-to-fuel efficiencies^16–19^.

Using metal-based (e.g., Au, Ag, Sn and Cu) catalysts^20–23^ or bacteria^12^, over 10-fold enhancement of the solar-to-fuel efficiencies compared to natural photosynthesis^24^ has been realized^12,17^. Nonetheless, to date, the highest reported solar-to-chemical efficiency^17^ of all solar-driven CO_2_ electrolyzers has remained at ~14%, with the highest electric energy efficiency below 50%. In addition, the operation of all systems still strongly relies on sunlight illuminations, which are altered significantly over time of a day, weathers and regions. Thus, designing artificial photosynthetic devices capable of highly selective CO_2_ reduction reaction (CO_2_RR) under different sunlight conditions is of crucial significance.

In green plants, natural photosynthesis is consisted of two main steps^25^, known as the light reaction and light-independent carbon fixation (i.e., dark reaction). Light reaction drives water oxidation and energy storage in adenosine triphosphate (ATP) and nicotinamide adenine dinucleotide phosphate (NADPH). In carbon fixation, the captured atmospheric CO_2_ is converted to sugars at the presence of ATP and NADPH, which act as redox media to further transfer energy into chemical bonds. Herein, we propose to introduce a redox medium to assist the energy transfer between artificial light reaction (i.e., water oxidation) and carbon fixation, which allows for flexible storage and release of the photogenerated electrons. The artificial light reaction and carbon fixation are able to be operated in both light and dark conditions, endowing this redox-medium-assisted system with unique stability and high efficiency under various sunlight conditions.

## Results

### Design of the redox-medium-assisted system

**Fig. 1:**
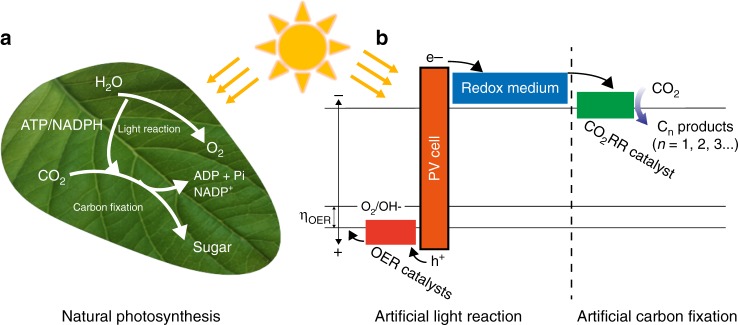
Schematic illustration of the redox-medium-assisted CO_2_ electroreduction system consisting of both light reaction and (dark) carbon fixation. **a** Reaction pathway of natural photosynthesis. **b** Energy diagram of each part in the redox-medium-assisted system

In order to mimic the two-step natural photosynthesis (Fig. 1a), we introduced a zinc/zincate (Zn/Zn(II)) redox pair to store the photogenerated electrons in the step of light reaction, as the charging of battery (Fig. 1b). These photogenerated electrons, as the reduction energy, can be controllably released to spontaneously reduce CO_2_ in the following artificial carbon fixation step, as the battery discharging. Thus, this redox medium not only features a similar role as its natural counterparts of ATP and NADPH, but also enables decoupling of the carbon fixation from light reaction to proceed at a different time. Furthermore, this photosynthesis-battery design allows a unique tuning capability of the CO_2_ conversion activity and selectivity by the discharging current densities.

In this redox-medium-assisted system, nano-gold (Au) and nickel-iron (NiFe) hydroxides were employed as electrocatalysts for CO_2_RR and oxygen evolution reaction (OER), respectively. A GaAs solar cell was utilized to drive the uphill OER and electron storage in Zn/Zn(II). The further release of electrons proceeded without light and led to an unassisted CO_2_ electroreduction.

### Nano-Au electrocatalyst for CO_2_RR

**Fig. 2:**
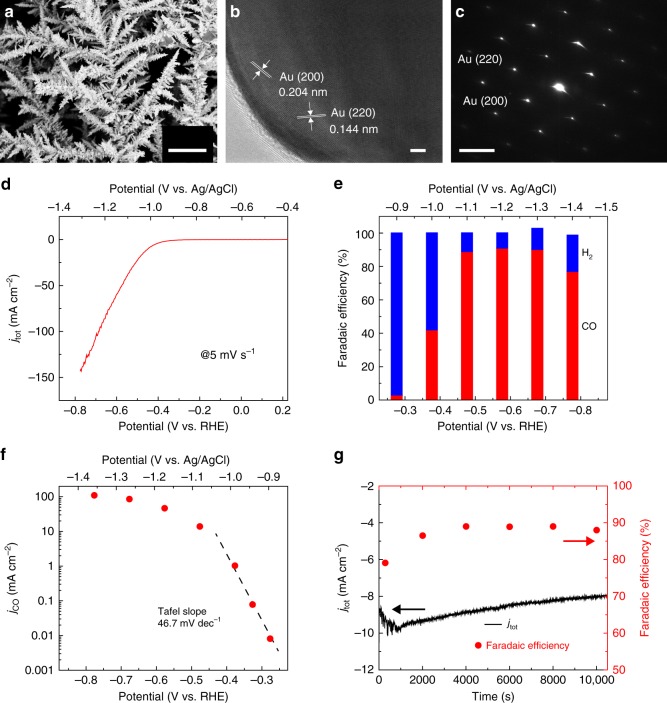
CO_2_ electroreduction on nano-Au catalysts. **a** SEM image of nano-Au catalysts on carbon papers. **b** HRTEM image and **c** SAED pattern of a representative nano-Au at the zone axis of <002 > direction. **d** Linear sweep voltammetry curve of nano-Au electrocatalysts in CO_2_-saturated 0.5 M KHCO_3_ solution at a sweep rate of 5 mV s^−1^. **e** Faradaic efficiency for CO (red bars) and H_2_ (blue bars) production on nano-Au at various potentials ranging from –0.27 to –0.77 V vs. RHE. **f** The current-voltage (*j*–*V*) curve of CO production partial current density versus potential on nano-Au and kinetics analysis of the CO_2_ electroreduction on nano-Au. **g** Stability of CO_2_ electroreduction activity of nano-Au at –0.47 V vs. RHE, including the total current density (left *y*-axis) and FE_CO_ (right *y*-axis). The scale bars are 10 μm in **a**, 2 nm in **b** and 5 1/nm in **c**

Nano-Au catalysts were grown on carbon paper via an electrodeposition method in the solution of HAuCl_4_ and HCl (Methods). The purity and crystalline structure of the obtained nano-Au were characterized by X-ray diffraction (XRD, Supplementary Figure 1). All the diffraction peaks were well indexed as cubic Au (JCPDS No. 04-0784). The obtained nano-Au catalyst exhibited a branched needle-like structure with several microns in length and high-curvature tips (Fig. 2a). High-resolution transmission electron microscopy (HRTEM) and selected-area electron diffraction (SAED) revealed the single-crystalline nature of the nano-Au (Fig. 2b, c). The well-resolved lattice spacings of 0.204 and 0.144 nm were consistent with the (200) and (220) planes in cubic Au^26^.

The electrocatalytic CO_2_ reduction on nano-Au was evaluated in a Nafion-membrane separated H-type electrochemical cell with CO_2_-saturated 0.5 M KHCO_3_ solution (pH 7.2) as the electrolyte (“Methods” section). The linear sweep voltammetry (LSV) profile of nano-Au exhibited a clear increase of total current density (*j*_tot_) corresponding to both CO_2_ and water reduction (Fig. 2d). The Faradaic efficiency for CO and H_2_ production (FE_CO_ and FE_H2_, respectively, Fig. 2e) were quantified by in-line gas chromatography. The FE_CO_ was started to be observed as ~ 3% at –0.27 V vs. reversible hydrogen electrode, RHE (equivalent to –0.9 V vs. Ag/AgCl), suggesting that the onset overpotential of CO_2_RR on nano-Au catalysts was less than 160 mV. With the increase of the applied potential from –0.27 to –0.77 V vs. RHE (equivalent to –0.9 to –1.4 V vs. Ag/AgCl), the yields of CO and H_2_ from CO_2_ and water reduction were both increased, manifested by the growing intensities of their gas chromatographic peaks (Supplementary Figure 2). However, the FE_CO_ exhibited a faster trend with the potential increase, peaking at –0.57 V vs. RHE (equivalent to –1.2 V vs. Ag/AgCl) with the maximum value of ~ 92% (Fig. 2e and Supplementary Figure 3). The product selectivity was also controlled by current density, as higher current densities were resulted from large overpotentials. As shown in Supplementary Figure 4, the Faradaic efficiency of CO was steady at ~90% when the current density was higher than 6 mA∙cm^−2^. No evident yield of liquid products was founded on the ^1^H nuclear magnetic resonance (NMR) spectra of the electrolyte after successive electrolysis at the potential range of –0.27 to –0.77 V vs. RHE for 20 h (Supplementary Figure 5).

The partial current density corresponding to CO_2_RR (*j*_co_) on nano-Au was shown in Fig. 2f, from which the Tafel analysis showed a slope of 47 mV∙dec^−1^. This value suggests a mechanism involving a fast-equilibrium of the formation of adsorbed CO_2_**·**^−^ intermediate and the following rate-determining formation of *COOH^1,27^. This needle-shaped nano-Au catalyst was previously proved to possess a high density of electric field at its tip, which was able to provide fast electron transfer during the first CO_2_ adsorption step^1,28^. Moreover, the nano-Au catalyst also exhibited a stable retention of FE_CO_ of ~ 90% at the potential of –0.47 V vs. RHE, with current retention of 80% after 10,000 s of continuous electrolysis (Fig. 2g).

### Water oxidation and Zn deposition

The artificial light reaction requires water oxidation on OER electrocatalysts and electron storage in a redox medium, which should possess a more negative thermodynamic equilibrium potential. NiFe hydroxide electrodes were synthesized by a hydrothermal method and investigated as anodes for O_2_ evolution (“Methods” section). The NiFe hydroxide grown on Ni foam exhibited a nanosheet morphology with a thickness of ~ 10 nm (Supplementary Figure 6). To evaluate its electrocatalytic OER performance, NiFe hydroxide electrodes were measured in a three-electrode setup with 1 M KOH aqueous solution. O_2_ evolution was observed at ~ 0.48 V vs. Ag/AgCl (equivalent to 1.50 V vs. RHE) with a current density of ~ 10 mA∙cm^−2^ (Supplementary Figure 7).

**Fig. 3:**
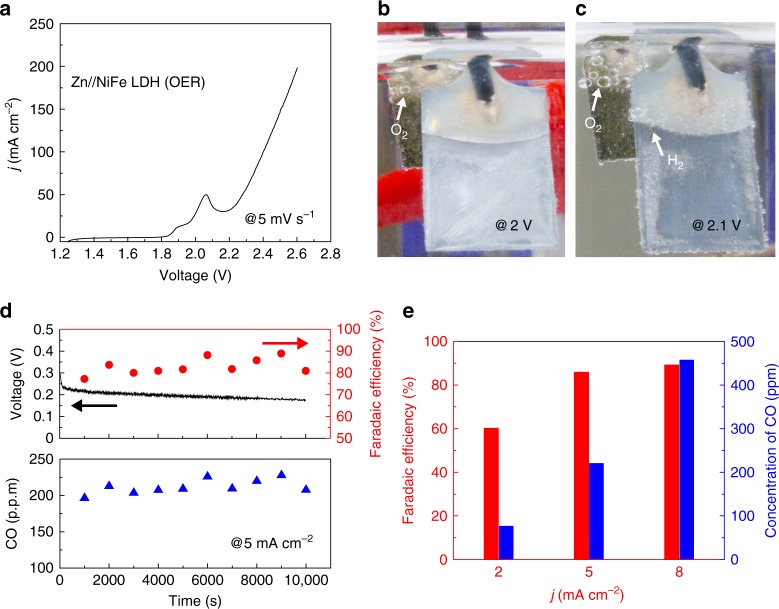
Oxygen evolution and CO_2_ reduction. **a** The *j*–*V* curve of electrochemical oxygen evolving in the two-electrode Zn//NiFe hydroxide system from linear sweep voltammetry. The scan rate was 5 mV∙s^−1^. **b**, **c** Photographs of hydrogen evolution on a Zn plate at a voltage of 2.0 and 2.1 V, respectively. **d** The chronovoltammetry profile, FE_CO_ and concentration of CO production in the two-electrode Zn//nano-Au system at a current density of 5 mA∙cm^−2^. **e** FE_CO_ (red bars) and concentration of CO production (blue bars) at different current densities of 2, 5 and 8 mA∙cm^−2^

Then, a two-electrode system was assembled with a NiFe hydroxide electrode as the anode and a Zn plate as cathode, using an electrolyte of 1 M KOH and 50 mM zinc acetate solution. The corresponding linear sweep voltammetry profile exhibited two adjacent peaks in the range of 1.8 to 2.1 V (Fig. 3a), ascribed to the oxidation of NiFe hydroxide anode and Zn deposition on the cathode, respectively^29^. H_2_ bubbles were not evidently observed on the Zn plate until the applied voltage was higher than 2.1 V (Fig. 3b, c), suggesting most of electrons were stored in the electrodeposited Zn instead of H_2_ from water reduction. The electrochemical potential for conducting the artificial light reaction was determined by quantifying the percentage of electrons contributing to Zn electrodeposition before prominent H_2_ evolution took place. The gaseous products on the cathode side at 2 V were analyzed by gas chromatography. The concentration of the obtained H_2_ on Zn cathode was ~ 5 parts per million (ppm), corresponding to a Faradaic efficiency of ~ 0.1% (Supplementary Figure 8). Thus, it confirms that at this potential, the electrodeposition of Zn predominates over the H_2_ evolution in the aspect of electron distribution during the artificial light reaction.

### Artificial carbon fixation

The artificial carbon fixation (electrocatalytic CO_2_ reduction) is indeed a variant form of the battery discharging. As shown in the Pourbaix diagram of Zn in aqueous electrolyte (Supplementary Figure 9), the divalent Zn mainly exists as Zn(OH)_4_^2−^ in 1 M KOH and 50 mM zinc acetate electrolyte (pH 14). The equilibrium potential of the Zn/Zn(OH)_4_^2−^ pair is –0.42 V vs. RHE at pH 14, which is more negative than that of the CO_2_ electroreduction to CO (–0.11 V vs. RHE) in the full pH range. The Zn/Zn(II) redox reaction is highly reversible (Supplementary Figure 10). Thus, electrons are able to spontaneously flow from Zn electrode toward nano-Au catalysts and reduce CO_2_ to CO.

By applying different circuit loads, three different current densities (i.e., 2, 5 and 8 mA∙cm^−2^) were applied to control the yield and selectivity of this process (Fig. 3d, upper panel, and Fig. 3e). All the energy was from the electrochemical energy of our system, and did not cost extra energy. A piece of bipolar membrane was employed to separate the neutral pH condition of CO_2_RR and the alkaline Zn/Zn(II) redox pair. The electrochemical reactions on each side are described by the following equations:

1 $${\mathrm{On}}\,{\mathrm{nano - Au}}\,{\mathrm{electrode:CO}}_{\mathrm{2}}{\mathrm{ + 2e}}^{\mathrm{-}}{\mathrm{ + 2H}}^{\mathrm{ + }} \to {\mathrm{CO + H}}_{\mathrm{2}}{\mathrm{O}}$$

2 $$\begin{array}{l}{\mathrm{On}}\,{\mathrm{Zn}}\,{\mathrm{electrode:Zn + 4OH}}^{\mathrm{-}} \to {\mathrm{Zn}}\left( {{\mathrm{OH}}} \right)_{\mathrm{4}}^{{\mathrm{2-}}}{\mathrm{ + 2e}}^{\mathrm{-}}\\ \left( {{\mathrm{by}}\,{\mathrm{taking}}\,{\mathrm{Zn}}\left( {{\mathrm{OH}}} \right)_{\mathrm{4}}^{{\mathrm{2-}}}{\mathrm{as}}\,{\mathrm{the}}\,{\mathrm{example}}} \right)\end{array}$$

3 $${\mathrm{At}}\,{\mathrm{the}}\,{\mathrm{bipolar}}\,{\mathrm{membrane:H}}_{\mathrm{2}}{\mathrm{O}} \to {\mathrm{H}}^{\mathrm{ + }}{\mathrm{ + OH}}^{\mathrm{-}}$$

At a current density of 2 mA∙cm^−2^, this artificial carbon fixation system yielded an average FE_CO_ of 60% with a mean voltage of ~0.33 V during 10,000 s of the reaction (Supplementary Figure 11a). With the current density increased to 5 mA∙cm^−2^, FE_CO_ was arisen to ~ 85% and remained stable, yielding an average CO concentration of ~ 220 ppm throughout the test with the same 10,000 s duration (Fig. 3d, bottom panel). The mean voltage of the corresponding chronovoltammetry curve was decreased to ~ 0.20 V as the consequence of increased overpotential loss at both the electrodes and bipolar membrane^16^. Further increasing the current density to 8 mA∙cm^−2^ resulted in a FE_CO_ of ~ 90%, and almost doubled CO concentration of 458 ppm in average (Fig. 3e and Supplementary Figure 11b). The FE values and concentration of CO for the artificial carbon fixation at each current density was summarized in Fig. 3e. It also demonstrates that this artificial carbon fixation system is capable of regulating the yield and component proportion of the CO_2_RR products without the assistance from extra bias voltage.

### Integrated redox-medium-assisted system

**Fig. 4:**
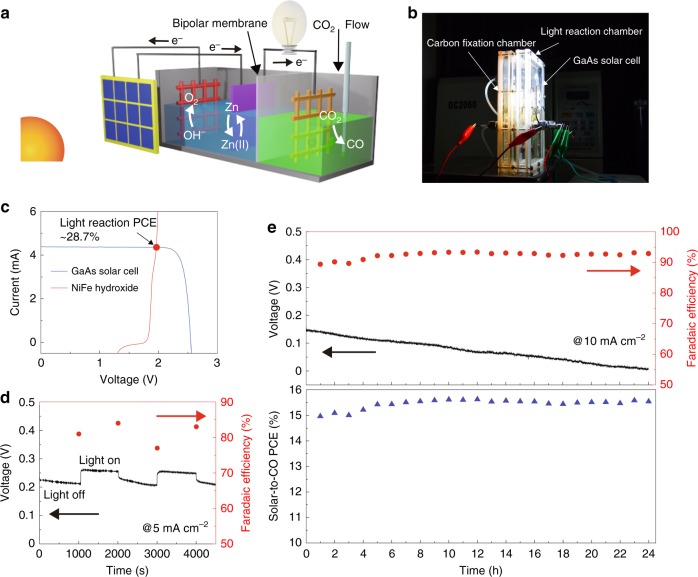
The redox-medium assisted CO_2_ electroreduction system. **a** Schematic and **b** photograph of the redox-medium-assisted system. **c** Photovoltaic and electrocatalytic current-voltage curves of the GaAs solar cell (blue curve) and two-electrode O_2_ evolution (red curve). The red dot indicates the maximum power conversion efficiency (PCE) point of the designed artificial light reaction, based on photoelectrochemical O_2_ evolution and Zn deposition. **d** Voltage-versus-time and FE_CO_ profiles of the overall photosynthesis under chopped simulated AM 1.5 G illumination. The current density for CO_2_ reduction was 5 mA∙cm^−2^. **e** The voltage-versus-time profile, FE_CO_, and solar-to-CO PCE of the redox-medium-assisted system at a current density of 10 mA∙cm^−2^

Finally, we assembled both the artificial light reaction (i.e., OER) and carbon fixation (i.e., CO_2_RR) devices into a redox-medium-assisted system. The schematic illustration and an optical photograph of this system are shown in Fig. 4a, b, respectively. A 0.5 × 0.5 cm^2^ triple-junction GaAs (InGaP/GaAs/Ge) solar cell was employed to harvest sunlight at standard air mass 1.5 G (Fig. 4c, blue curve), with a short circuit current (*i*_sc_) of 4.38 mA, an open circuit voltage (*V*_oc_) of 2.54 V, a maximum power (*P*_m_) of 9.49 mW, a fill factor (FF) of 85.2% and a photoconversion efficiency of 37.9% (Supplementary Figure 12). This GaAs solar cell was used to drive the OER and Zn deposition to complete the light reaction. During the artificial light reaction, the photogenerated holes flow to the surface of the NiFe hydroxide electrode and oxidize water into O_2_, whereas the photogenerated electrons reduce Zn(OH)_4_^2–^ at the surface of Zn plate. The Zn electrodeposition was confirmed by XRD when a Cu foam was used to substitute the Zn plate after 200 s of illumination (Supplementary Figure 13). By releasing electrons stored in Zn metals at different rates, the artificial carbon fixation exhibited a tunable selectivity and yield of CO_2_RR on the surface of nano-Au electrocatalysts. The unassisted artificial light reaction was operated at 1.96 V and 4.35 mA (Fig. 4c, indicated by the red dot). Based on the thermodynamic equilibrium potential of water oxidation (1.23 V vs. RHE) and Zn electrodeposition (–0.42 V vs. RHE)^17^, the theoretical voltage of the artificial light reaction is 1.65 V. According to Eq. 6 in the “Methods” section (also shown in Supplementary Table 1), the free-energy conversion efficiency (also known as power conversion efficiency) of the light reaction presented a maximum value of ~ 28.7%.

To investigate the influence of illumination intensity to FE_CO_, this redox-medium-assisted system was tested under a chopped sunlight illumination with a duration of 1000 s for each light and dark measurement (Fig. 4d). Throughout the test at the current density of 5 mA∙cm^−2^ for carbon fixation, the FE_CO_ remained relatively stable at 82 ± 4%, and the only effect of the chopped sunlight was the synchronously changed voltage. Under 1-sun illumination, the current for light reaction was higher than that for carbon fixation (Supplementary Figure 14), making Zn electrodeposition predominates over its oxidation, leading to a more negative potential on Zn plate and increased carbon fixation voltage.

The long-term solar-driven CO_2_ elelctroreduction was measured at different current densities for electron releasing during the carbon fixation. The current density determines the proportion of the stored electrons consumed by CO_2_RR and HER on nano-Au. For instance, at a 10 mA∙cm^−2^ current density (i.e., ~ 3.2 mA), ca. 73.5% of the stored electrons participate the carbon fixation reaction. According to Equation 4 in the “Methods” section, the solar-to-CO PCE (PCE_chem_) of the whole system was determined by the Faradaic efficiency of Zn(II) reduction and CO formation, and the utilization of stored electrons during the carbon fixation process (i.e., the current density of CO_2_RR). The maximum current density for the CO_2_RR in this photosynthesis-mimicking cell was ~ 13.5 mA∙cm^−2^ (i.e., 4.35 mA), which was gradually decreased during the operation of 600 s (Supplementary Figure 15).

At the current density of 5 mA∙cm^−2^, about one third of the stored photogenerated electrons were released during the carbon fixation, in which FE_CO_ was steady at ~ 87% for over 90 h, leading to a solar-to-CO PCE of ~ 7.2% (Supplementary Figure 16). The energy loss in carbon fixation was attributed to the combined effect of the thermodynamic difference between light reaction (1.65 V) and the overall process (1.34 V), the overpotential of each electrochemical reaction, the internal resistance of the device (~ 56 ohm, Supplementary Figure 17), and the energy consumed by water dissociation on bipolar smembrane (~ 0.13 V, Supplementary Figure 18).

By increasing the current density to 10 mA∙cm^−2^, the output voltage of the carbon fixation system was decreased to ~ 0.14 V and eventually close to 0 V after 24 h of continuous test (Fig. 4e, upper panel). The gradual voltage decay was attributed to the overpotential increase with time, which was more significant at higher current densities. On average, an FE_CO_ of 92% was obtained, with a high stability over the course of measurement (Fig. 4e, lower panel). At a current density of 10 mA∙cm^−2^ (i.e., 3.2 mA), the light reaction operating voltage was constantly 1.96 V (Supplementary Table 1); the thermodynamic voltage of the overall reaction was 1.34 V; the potential on Au electrode was –1.1 V vs. Ag/AgCl; the potential on Zn was –1.38 V vs. Ag/AgCl; and membrane voltage was ~ 0.13 V. According to Equation 7 in the Methods section, the corresponding solar-to-CO efficiency was calculated to be as high as 15.6%, which to the best of our knowledge, represents the highest solar-to-chemical efficiency reported to date. This efficiency substantially surpasses the previous record of the solar-to-chemical efficiency (13.8%) by using a bifunctional SnO_2_/CuO catalyst powered by a multi-junction GaInP/GaInAs/Ge photovoltaic^17^.

For comparison, when this system was only assembled with the nano-Au catalyst cathode and NiFe hydroxide anode, but without the Zn/Zn(II) redox medium, the solar-to-CO PCE reached 16.4% with an operating voltage of 2.4 V (Supplementary Figure 19). Although our two-step solar-driven CO_2_ electroreduction system presented a slightly lower solar-to-chemical efficiency (i.e., 15.6%), it allowed for a much improved operating voltage and excellent FE stability to the ever-changing sunlight illumination.

## Discussion

The key to the design of this redox-medium-assisted system exists in the rational design of catalysts, redox media and the bipolar membrane. The Zn/Zn(II) redox pair is employed due to its more negative equilibrium potential in comparison to the potential of CO_2_ reduction on the nano-Au catalysts. The bipolar membrane allows the use of both the alkali Zn/Zn(II) pair and the close-to-neutral pH electrolyte for CO_2_RR. In addition, compared to the conventional solar-driven electrolysis for CO_2_RR of which the selectivity is controlled by the operating voltage^17,19^, our design exhibits a current-controlled Faradaic and power conversion efficiency, capable of a lower voltage demand and high catalytic performances. Compared to the direct solar cell (or battery)-driven electrolysis (Supplementary Figure 19), our two-step system exhibits a lowered operation voltage of 1.96 V at 10 mA∙cm^−2^ with a FE_co_ of ~ 92%, yielding an outstanding electric energy efficiency of ~ 63% (Supplementary Figure 20), which is comparable to the best reported efficiency of 64% by a Au nanoneedle//NiCoFeP system^30^. To improve the current density, this device architecture was combined with a gas-diffusion-layer (GDL)-based flow electrolyzer and a Si solar cell. The current density of the carbon fixation was boosted to 100 mA∙cm^−2^ (Supplementary Figure 21). At this current density, the system exhibited a CO Faradaic efficiency of ~ 80%, an electric energy efficiency of ~48%, and a solar-to-CO efficiency of ~ 5%, thus suggesting the potential of scalable production of renewable fuels in the future.

Moreover, our design endows the artificial photosynthesis with high efficiency, tunable selectivity, excellent stability and flexibility to various sunlight conditions even in the dark, owing to the independent processes of the light reaction and carbon fixation. In this regard, our two-step, redox-medium-assisted electrolyzer system presents clear advances in the PCE and stability under the ever-changing sunlight illumination. The present work utilized noble metal catalysts as a demonstration. Lowering the cost by using earth-abundant metal catalysts with rational material engineering is certainly the direction toward practical applications. Combining with further electrocatalyst development toward other CO_2_ reduction products such as CH_4_, C_2_H_4_ and alcohol, as well as investigating new redox media capable of providing higher potential difference for carbon fixation, our design may further enable exciting advantages to the highly stable and efficient solar energy-driven electrochemical synthesis of value-added chemicals/fuels from CO_2_.

In summary, we present a two-step, a redox-medium-assisted solar-driven CO_2_ electroreduction system design for efficient electrocatalytic CO_2_RR, by incorporating a Zn/Zn(II) redox medium to mimic the functionality of ATP/ADP that acts as the electron carrier during the natural photosynthesis. In the light reaction, the solar-driven oxygen evolution and Zn(II) reduction store electrons in the Zn/Zn(II) medium. The carbon fixation releases the stored electrons and leads to an unassisted electrochemical reduction of CO_2_. We achieved an excellent electric energy efficiency of 63%, and a record-high solar-to-CO efficiency peaking at 15.6% under 1-sun intensity. Our system further allows the tuning of activity and selectivity of this CO_2_ electrocatalysis, suggesting new opportunities in manufacture of non-renewable fossil fuels from CO_2_ and sunlight in such a redox-medium-assisted approach.

## Methods

### Preparation of nano-Au electrode

The nano-Au electrode was prepared through three steps: electrodeposition, purification and spreading. First, nano-Au was electrodeposited at a constant potential of –0.4 V (vs. Ag/AgCl) for 300 s on a carbon paper (Toray TGP-H-060), in the solution consisting of 0.5 M HCl and 0.16 M HAuCl_4_·3H_2_O ( ≥ 49.0% on Au basis, Sigma-Aldrich). To facilitate close contact between the catalysts and carbon paper substrate during the electrocatalytic process, nano-Au was peeled from the substrate by ultrasonication, mixed with Nafion solution and spread on another fresh carbon paper (0.25–0.35 cm^2^).

### Preparation of NiFe hydroxide electrode

The NiFe hydroxide electrode was synthesized on a Ni foam via a hydrothermal method. In brief, 40 mL of aqueous solution containing 0.5 mmol Ni(NO_3_)_2_·6H_2_O, 0.5 mmol Fe(NO_3_)_3_·9H_2_O, and 5 mmol CO(NH_2_)_2_ were transferred into a 50-mL autoclave with a piece of Ni foam (~ 5 cm^2^). The NiFe hydroxides were grown on Ni foam via the hydrothermal reaction at 120 °C for 12 h. Then, the NiFe hydroxide electrode was rinsed with water and ethanol, dried at 60 °C, and cut into small piece with an area of 0.25–0.35 cm^2^.

### Electrocatalytic measurement of CO_2_ reduction on nano-Au

The electrocatalytic measurements were carried out in a gas-tight H-type electrolytic cell using a three-electrode system without *iR* compensation, which was connected with an electrochemical workstation (Autolab PGSTAT302). The H-type electrolytic cell was comprised of two chambers separated by an ion-exchange membrane (Nafion117). The nano-Au catalyst, Ag/AgCl electrode (3.5 M KCl used as the filling solution) and a platinum (Pt) wire were employed as working, reference and counter electrodes, respectively. Before testing, the electrodes were dried at 80 °C in vacuum overnight to ensure no residual isopropanol was brought into the electrolyte. The observed potentials were converted to reversible hydrogen electrode (RHE) scale through the following formula:

4 $${{E}}_{{\mathrm{RHE}}} = E_{{\mathrm{Ag/AgCl}}\,{\mathrm{(3}}{\mathrm{.5}}\,{\mathrm{M}}\,{\mathrm{KCl)}}}{\mathrm{ + 0}}{\mathrm{.059}} \ast {\mathrm{pH + 0}}{\mathrm{.205}}$$

A 0.5 M CO_2_-saturated KHCO_3_ (pH 7.2) was used as the electrolyte in both of the anodic and cathodic chambers. Catholyte was stirred at the rate of 1700 rounds per minute (r.p.m.) throughout the electrolysis. The flow rate of the CO_2_ gas transporting into the cathodic chamber was fixed at 30 standard cubic centimeters per minute (s.c.c.m). The gaseous products of CO_2_ reduction reaction were separated by gas chromatography, and detected by a thermal conductivity detector (TCD) and a flame ionization detector (FID). High-purity Argon (99.99%) was used as the carrier gas throughout the separation and analysis. Liquid products were quantified by ^1^H nuclear magnetic resonance (NMR, Bruker DRX 500). Faradaic efficiency for product *x* (FE_*x*_) was calculated based on the following equation:

5 $${\mathrm{FE}}_x{\mathrm{ = }}\frac{{i_x}}{{i_{{\mathrm{tot}}}}}{\mathrm{ = }}\frac{{n_xv_{{\mathrm{gas}}}c_xF}}{{i_{{\mathrm{tot}}}V_m}},$$

where *i*_*x*_ represents the partial current of product *x*; *i*_tot_ is the overall current; *n*_*x*_ represents the number of electron transfer towards the formation of 1 mol of product *x*; *v*_gas_ denotes the flow rate of CO_2_ (s.c.c.m); *c*_*x*_ denotes the concentration of product *x* detected by the gas chromatography (p.p.m); *F* is the Faraday constant (96,485 C∙mol^−1^); *V*_m_ represents unit molar volume, which is 24.5 L·mol^−1^ at the room temperature (298.15 K).

### Measurements of water oxidation and Zn deposition

The electrochemical water oxidation on NiFe hydroxide electrodes was tested in a three-electrode system in an electrochemical workstation (Autolab PGSTAT302) without *iR* compensation. 1 M KOH was used as the electrolyte. To test the faradaic efficiency of Zn electrodeposition, a sealed H-type electrolytic cell separated by Nafion117 membrane was employed, filling with a mixed solution containing 1 M KOH and 50 mM zinc acetate. Zn plates (1 cm^2^), NiFe hydroxides and Ag/AgCl electrode (3.5 M KCl used as the filling solution) were used as working, counter and reference electrodes, respectively. The faradaic efficiency of Zn electrodeposition was obtained by the calculation and subtraction of hydrogen evolution reaction faradaic efficiency on Zn plates via gas chromatography.

### The redox-medium-assisted system

The redox-medium-assisted CO_2_ electroreduction was carried out in a homemade cell containing three parts: a photovoltaic cell, a light reaction chamber, and a carbon fixation chamber. A triple-junction GaAs (InGaP/GaAs/Ge) solar cell (0.5 × 0.5 cm^2^) was purchased from Qiansui Trading Co., Ltd. (Shanghai, China), and adopted in this system for the harvesting of solar energy. The light reaction and carbon fixation chambers were separated by a bipolar membrane (Fumasep FBM-Bipolar, Fuel Cell Store). 1 M KOH with 50 mM zinc acetate (pH 14) and 0.5 M CO_2_-saturated KHCO_3_ (pH 7.2) were used as electrolytes for light reaction and carbon fixation, respectively. The NiFe hydroxide electrode and Zn plate were driven by the GaAs solar cell under standard air mass 1.5 G solar light at room temperature. For the artificial carbon fixation, the nano-Au electrode and Zn plate were employed as the cathode and anode, respectively. The test was operated via chronovoltammetry method at a current density of 2, 5 and 8 mA∙cm^−2^, respectively. To improve the current density, gas diffusion layers (GDL, Freudenberg H14C9, 1.5 × 1.5 cm^2^) were used to spray coat nano-Au catalysts with a mass loading of 0.5 mg∙cm^−2^. In this case, high-concentration electrolytes were utilized to reduce the *iR* loss and boost the current density for both light and carbon reactions. A 25 cm^2^ Si solar cell (Ningbo Xinghui Xuanyang Energy & Technology Co., Ltd., China) was used to power the system. The analysis of CO_2_ reduction product and the subsequent calculation were conducted through the same procedure of the electrocatalytic testing of CO_2_RR. The light reaction efficiency (PCE_light_) and the solar-to-chemical efficiency (PCE_chem_) are defined as the ratios of the stored solar power in Zn (*P*_light_) and fuel energy (*P*_chem_) vs. the solar power (*P*_solar_), respectively, calculated from the following equations:

6 $${\mathrm{PCE}}_{{\mathrm{light}}}{\mathrm{ = }}\frac{{P_{{\mathrm{light}}}}}{{P_{{\mathrm{solar}}}}}{\mathrm{ = }}\frac{{E_{OER + Zn}^0{\mathrm{FE}}_{{\mathrm{Zn}}}i_{{\mathrm{light}}}}}{{P_{{\mathrm{solar}}}A_{{\mathrm{solar}}}}}$$

7 $${\mathrm{PCE}}_{{\mathrm{chem}}}{\mathrm{ = }}\frac{{P_{{\mathrm{chem}}}}}{{P_{{\mathrm{solar}}}}}{\mathrm{ = }}\frac{{E_{co}^0{\mathrm{FE}}_{{\mathrm{Zn}}}{\mathrm{FE}}_{ch{\mathrm{em}}}i_{ch{\mathrm{em}}}}}{{P_{{\mathrm{solar}}}A_{{\mathrm{solar}}}}},$$

where *P*_light_ and *P*_chem_ represent the power used for the artificial light reaction and the carbon fixation, respectively; *P*_solar_ stands for the overall solar power input, which is 100 mW cm^−2^; *E*^0^_co_ represents the equilibrium potential difference between the CO_2_ electroreduction to CO and the water oxidation to O_2_ (1.34 V); *A*_solar_ is the geometric area of the three junction GaAs solar cell (0.25 cm^2^). FE_Zn_ is the Faradaic efficiency for Zn electrodeposition during light reaction, which is 1 for direct solar-driven CO_2_RR; FE_chem_ is the Faradaic efficiency for the artificial carbon fixation; *i*_light_ and *i*_chem_ are the currents during the light reaction and carbon fixation, respectively.

The electric energy efficiency was defined as the ratio of fuel energy to applied electric energy, which was calculated with the following equation:

8 $${\mathrm{Electrical}}\,{\mathrm{energy}}\,{\mathrm{efficiency = }}\frac{{E_{{\mathrm{chem}}}}}{{E_{{\mathrm{applied}}}}}{\mathrm{ = }}\frac{{E_{{\mathrm{co}}}^0{\mathrm{FEZnFE}}_{ch{\mathrm{em}}}}}{{{\mathrm{Applied}}\,{\mathrm{Voltage}}}},$$

where *E*_chem_ stands for the energy used for the artificial carbon fixation; *E*_applied_ stands for the input electrical energy; *E*^0^_co_ represents the equilibrium potential the driven reaction which is 1.34 V for CO_2_RR. FE_Zn_ is the Faradaic efficiency for Zn electrodeposition, which is 1 for system without Zn/Zn(II) pairs; FE_chem_ is the Faradaic efficiency of the artificial carbon fixation.

## Electronic supplementary material

Supplementary Information

## Data Availability

The data sets generated and/or analyzed during the current study are available from the corresponding author on reasonable request.
