# Supplementary material for: Efficient solar-driven electrocatalytic CO2 reduction in a redox-medium-assisted system
Source: Nat Commun. 2018 Nov 27;9:5003. doi: 10.1038/s41467-018-07380-x (PMC6258760; doi:10.1038/s41467-018-07380-x)
Supplement: Supplementary file 1 — Supplementary Information [file 41467_2018_7380_MOESM1_ESM.pdf]

# Efficient solar-driven electrocatalytic $CO_2$ reduction in a redox-medium-assisted system

Yuhang Wang,[†] Junlang Liu,[†] Yifei Wang, Yonggang Wang, Gengfeng Zheng*

## Supplementary Figures

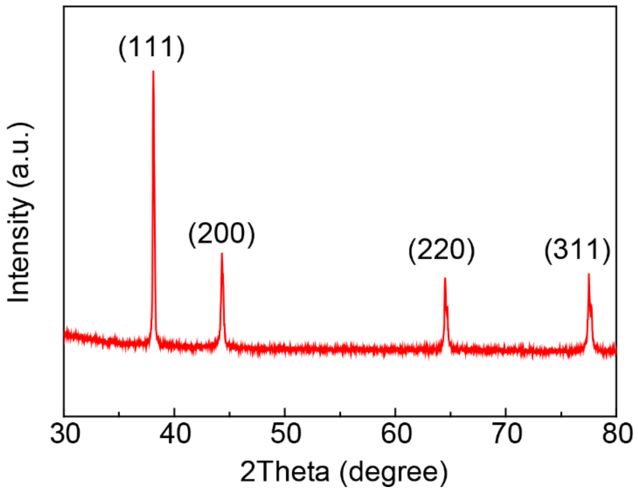


**Supplementary Figure 1.** XRD pattern of the as-synthesized nano-Au on carbon paper.

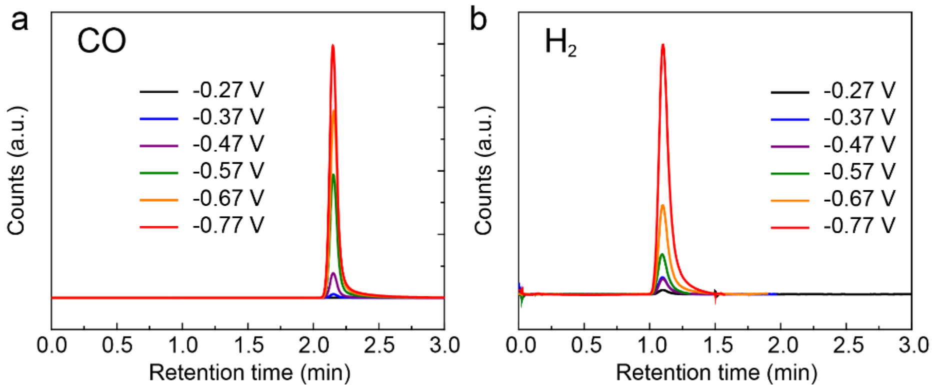


**Supplementary Figure 2. Detection of $CO_2$RR products.** Chromatograms of **a** CO and **b** $H_2$ at various applied potentials ranging from –0.27 to –0.77 V vs. RHE.

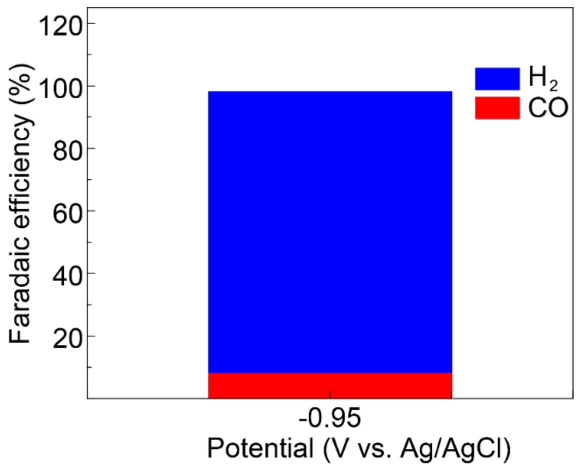


**Supplementary Figure 3.** Faradaic efficiencies of CO (red bars) and $H_2$ (blue bars) on nano-Au at a potential of –0.95 V vs. Ag/AgCl (i.e., –0.33 V vs. RHE).

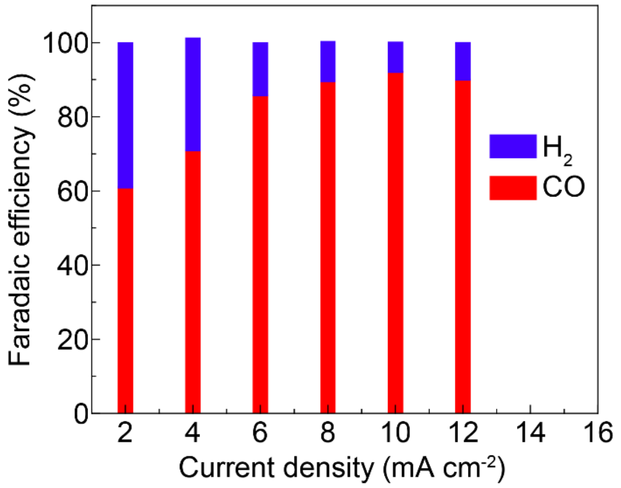


**Supplementary Figure 4.** Faradaic efficiency for CO (red bars) and $H_2$ (blue bars) production on nano-Au at various potentials ranging from 2 to 12 mA $cm^{-2}$.

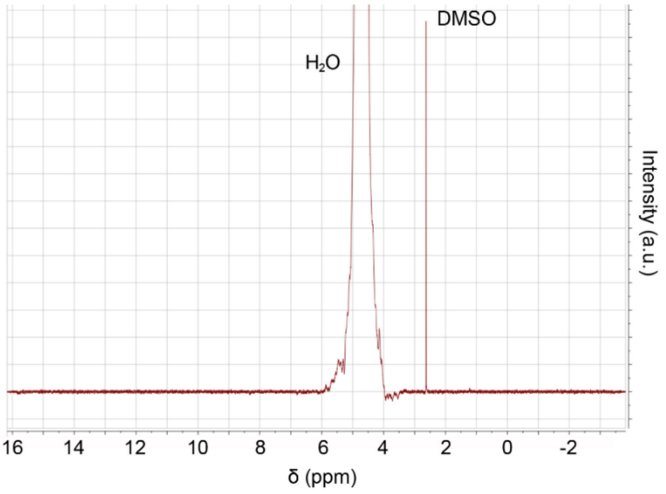


**Supplementary Figure 5**. Representative H-NMR spectrum of the electrolyte after successive electrochemical $CO_2$ reduction for 20 h, showing no other liquid products. Peaks present here were from $H_2O$ and DMSO that were used as the internal standards.

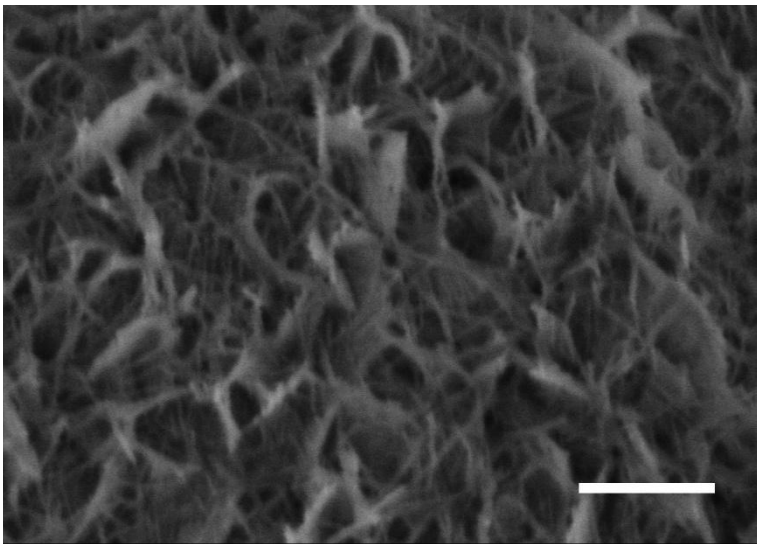

**Supplementary Figure 6.** SEM image of NiFe hydroxides grown on Ni foam. The scale bar is 200 nm.

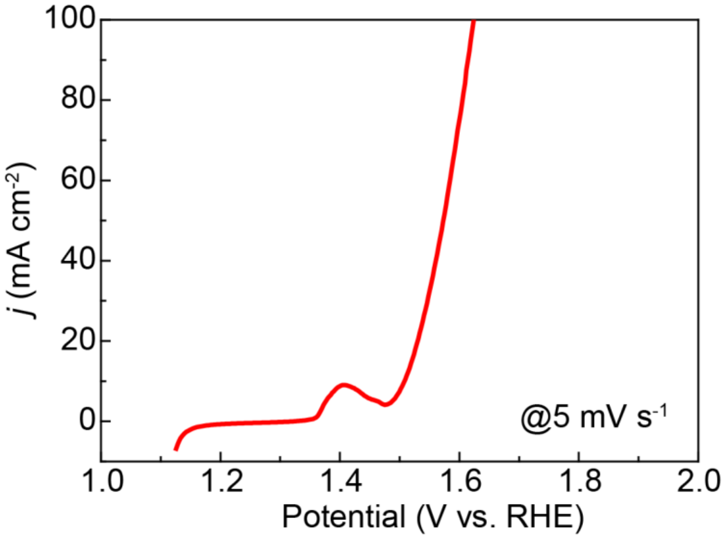


**Supplementary Figure 7.** Linear sweep profile of OER on NiFe hydroxides at 5 mV $s^{-1}$.

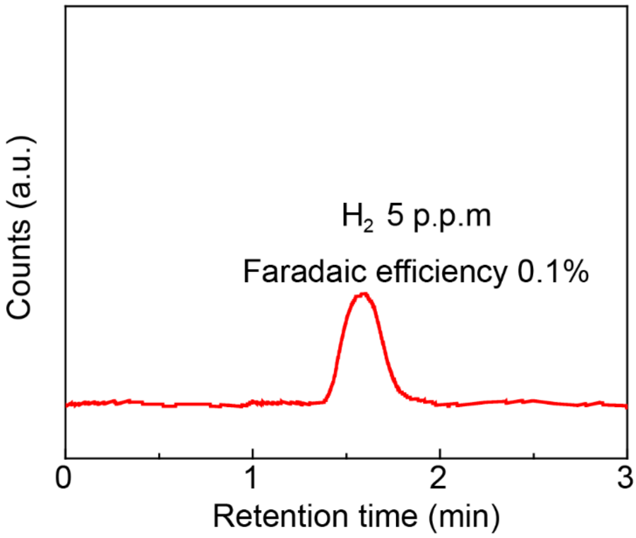


**Supplementary Figure 8.** Gas chromatographic analysis of the $H_2$ evolution on the Zn plates during the electrodeposition of Zn at an applied voltage of 2 V.

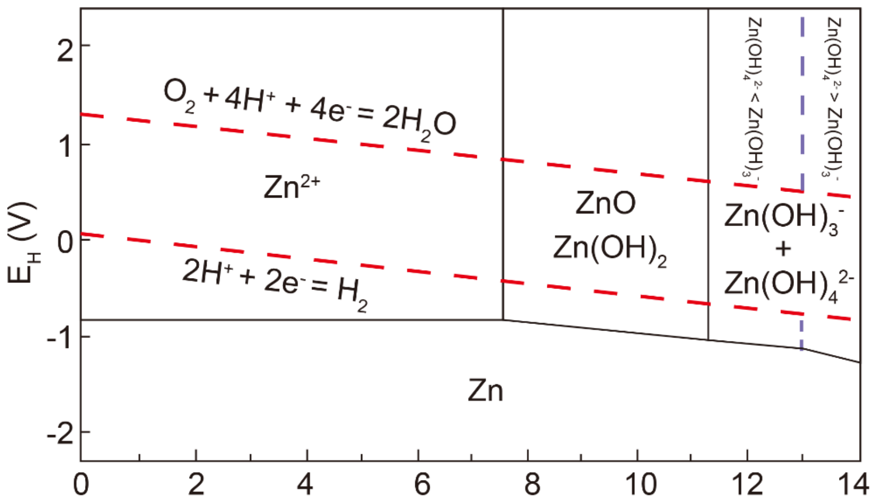


**Supplementary Figure 9.** Pourbaix diagram of Zn in aqueous electrolyte (drawn by the authors according to literatures[1, 2]). In 1 M KOH (pH 14), the divalent Zn is mainly in $Zn(OH)_4^{2-}$ form.

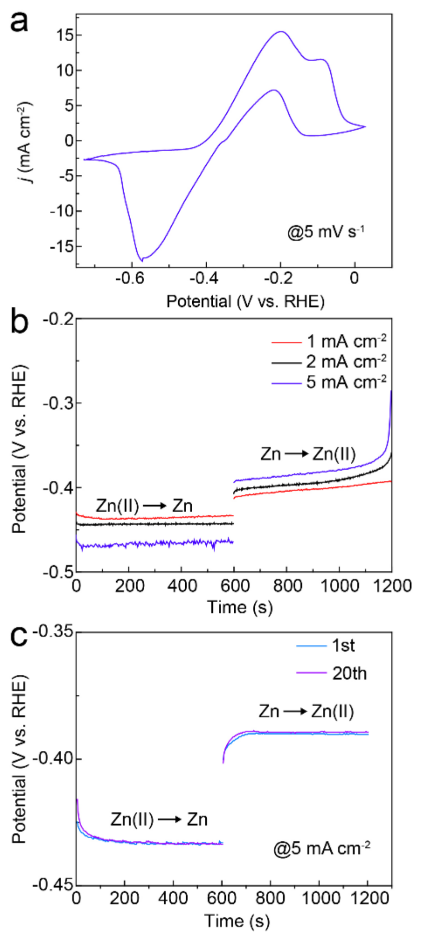


**Supplementary Figure 10. The electrochemical reversibility of the Zn/Zn(II) medium. a** Cyclic voltammetry profile of Zn/Zn(II) redox pair in 1 M KOH. The anodic peak at -0.22 V was the oxidation of zinc to $Zn(OH)_4^{2-}$, and peak at -0.13 V to $Zn(OH)_3^-$ was due to the depletion of $OH^-$ in solution near the electrode surface[2]. **b** Charge-discharge profile of Zn/Zn(II) half-cell reaction on a Cu foil substrate at different current density with the same charge-discharge time. **c** Charge-discharge profile of Zn/Zn(II) half-cell reaction on a Zn foil substrate with a cut-off capacity of 0.83 mA h at the current density of 5 mA $cm^{-2}$. The profile on Zn foil exhibited a higher stability versus time compared to the voltage on Cu foil. The Faradaic efficiency (or Coulombic efficiency) of the reaction in both cases were ~ 100%, indicting the highly reversible nature of this redox pair.

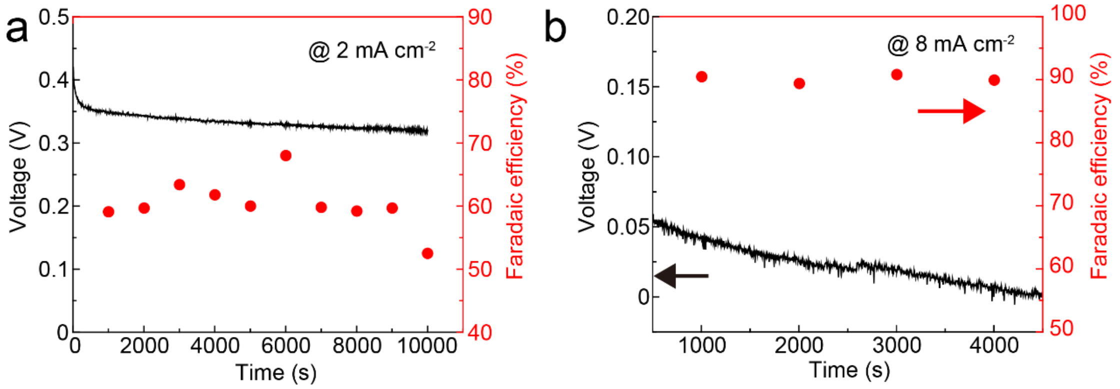


**Supplementary Figure 11. Carbon fixation efficiency at different current density.** Chronovoltammetry profile (left *y*-axis) and $FE_{CO}$ (right *y*-axis) of the artificial carbon fixation in the two-electrode Zn//nano-Au system at a current density of **a** 2 and **b** 8 mA $cm^{-2}$.

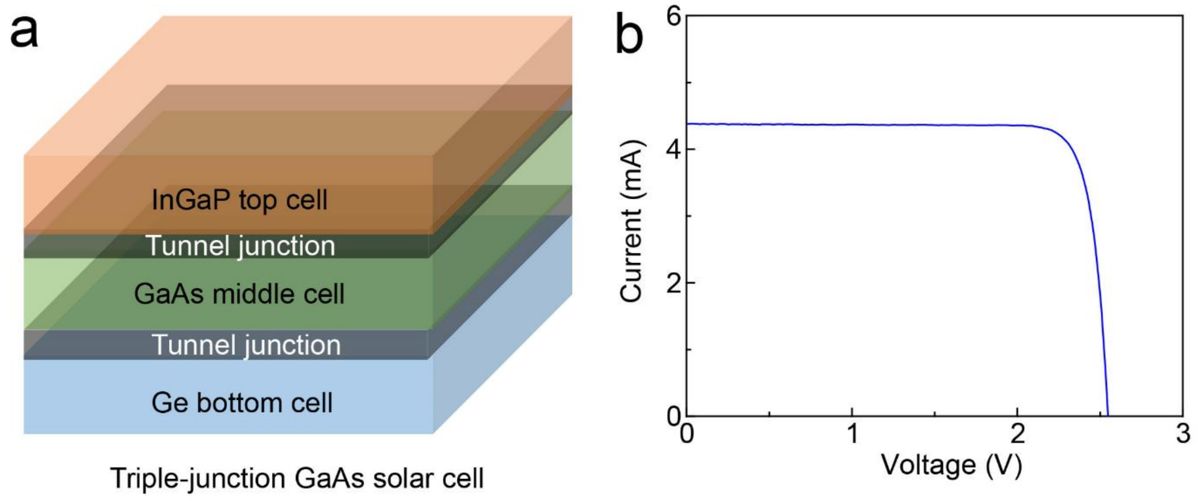


**Supplementary Figure 12. The triple-junction solar cell. a** Structure illustration and **b** photovoltaic current-voltage curves of the triple-junction InGaP/GaAs/Ge solar cell.

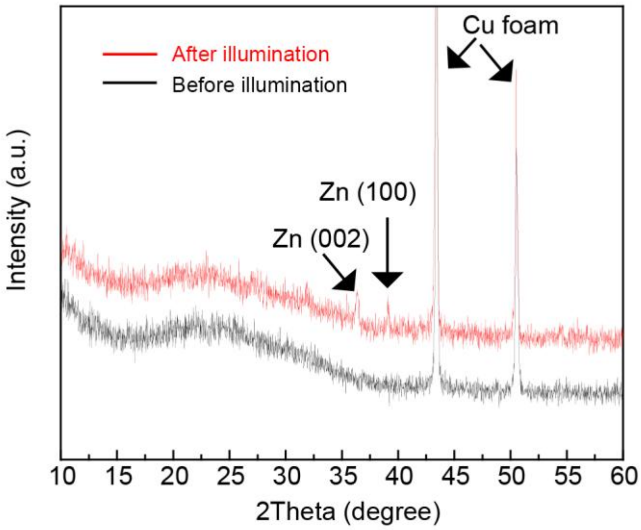


**Supplementary Figure 13.** XRD patterns of a Cu foam before and after 200 s of sunlight illumination. The Cu foam was employed as the substitution of Zn plates as the means of revealing the Zn electro-deposition during the light reaction.

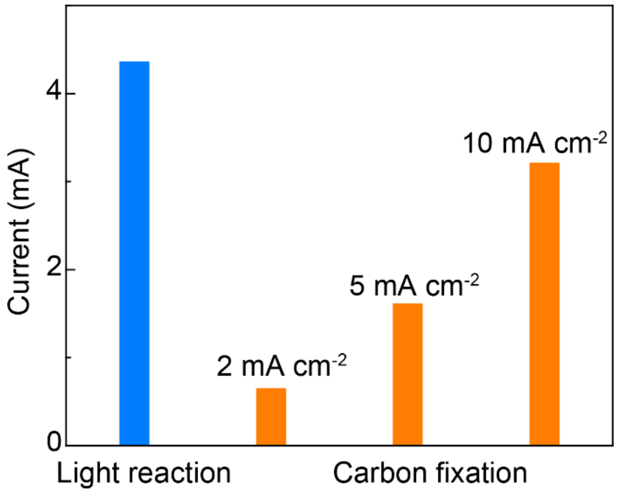


**Supplementary Figure 14.** Comparison of light reaction and carbon fixation current.

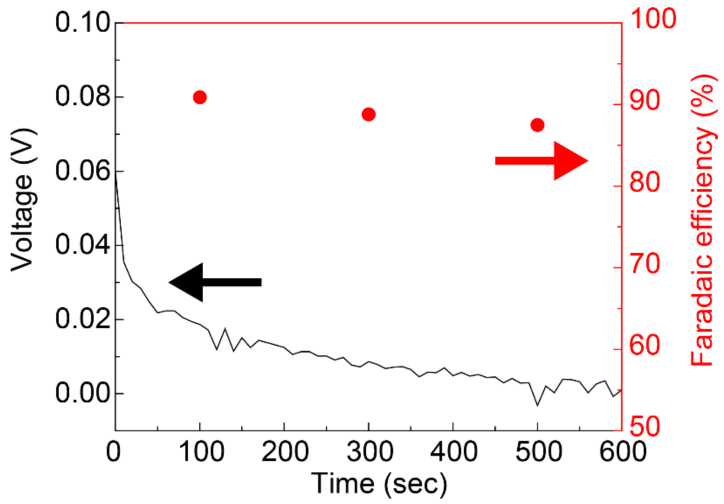


**Supplementary Figure 15.** Chronovoltammetry profile (left *y*-axis) and $FE_{CO}$ (right *y*-axis) of the artificial photosynthesis-battery system at a current density of 13.5 mA $cm^{-2}$ (i.e., 4.35 mA) under 1-sun illumination. The quick voltage decay at 4.35 mA was assigned to the combined effect of overpotential, *iR* increase, catalyst decay at high current, and the fast discharge of the double layer in the first few seconds. The upper cap of the photocurrent of the triple-junction InGaP/GaAs/Ge solar cell in this carbon-paper-based system was 4.35 mA, which was also the highest current for carbon fixation before the Zn foil substrate was consumed

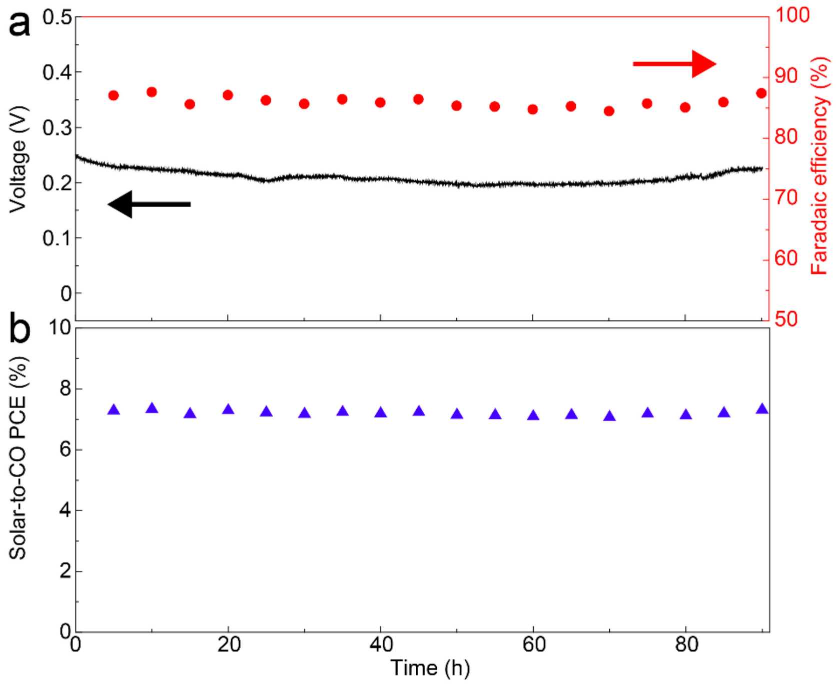


**Supplementary Figure 16. Redox-medium-assisted system at 5 mA cm$^{-2}$. a** Chronovoltammetry profile (left *y*-axis) and $FE_{CO}$ (right *y*-axis) of the artificial photosynthesis-battery system at a current density of 5 mA cm$^{-2}$ under 1 sun illumination. **b** The corresponding solar-to-CO PCE over the course of operation.

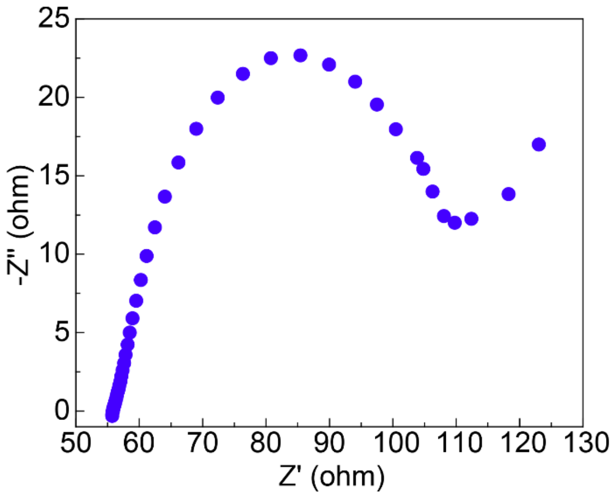


**Supplementary Figure 17.** Electrochemical impedance spectroscopy of the carbon fixation system. The internal resistance was calculated as ~ 56 ohm.

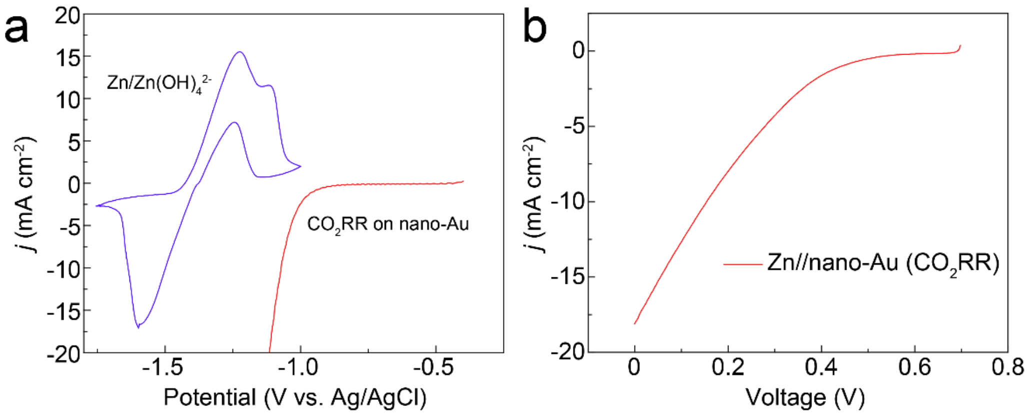


**Supplementary Figure 18. Determination of *iR* loss on bipolar membrane. a** Cyclic voltammetry profile of $Zn/Zn(OH)_4^{2-}$ and linear sweep profile of $CO_2RR$ on nano-Au catalysts at 5 mV $s^{-1}$. **b** Linear sweep profile of the carbon fixation Zn//nano-Au system at 5 mV $s^{-1}$. Considering the area of Zn plate (~ 1 $cm^2$)and nano-Au electrode (~ 0.32 $cm^2$), for a $CO_2RR$ current density of 10 mA $cm^{-2}$, the potential vs. Ag/AgCl on each side is –1.4 and –1.1 V, respectively. It means when the current density is 10 mA $cm^{-2}$ on nano-Au electrode, a 0.27 V voltage could be obtained. However, the actual voltage of the system at 10 mA $cm^{-2}$ was ~ 0.14 V, indicating a 0.13 V *iR* loss on bipolar membrane.

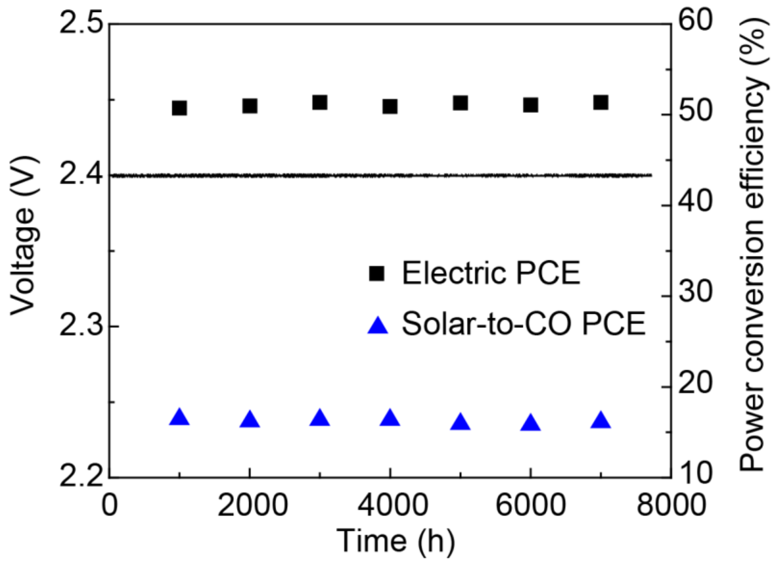


**Supplementary Figure 19.** The voltage-versus-time profile (left *y*-axis) and power conversion efficiency (right *y*-axis) of the sunlight-driven electrochemical $CO_2$RR device, ***without*** the Zn (II)/Zn redox pair.

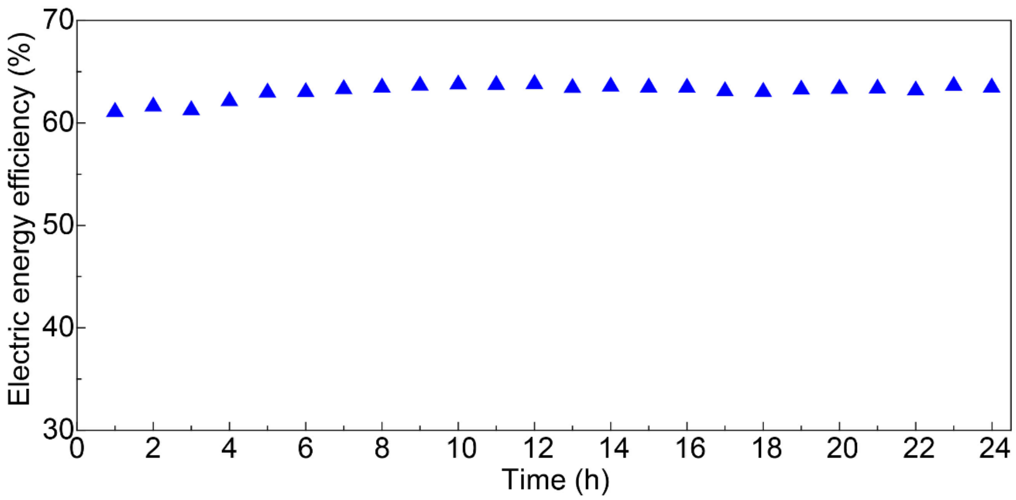


**Supplementary Figure 20.** Electric energy efficiency (Electric PCE) of the photosynthesis-battery system at a current density of 10 mA·cm$^{-2}$.

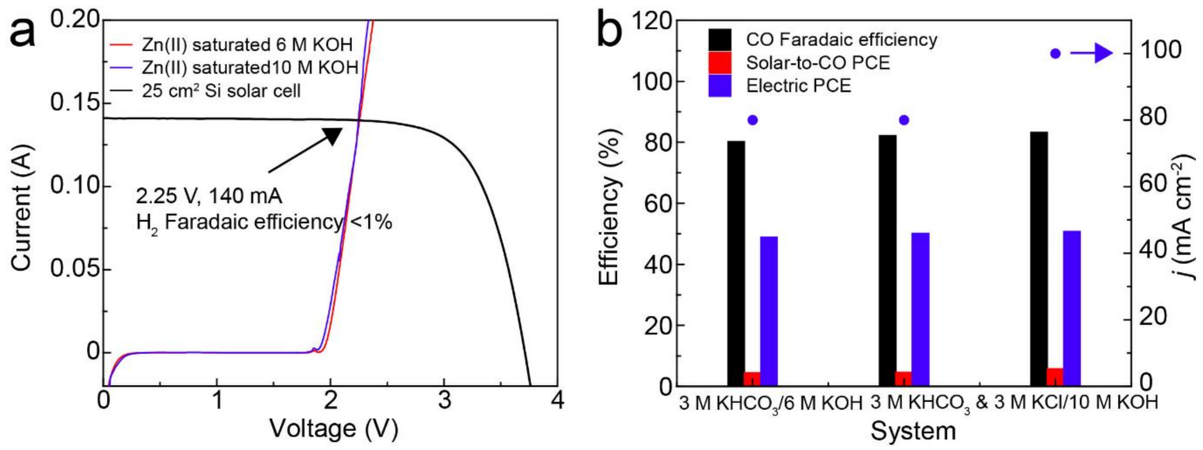


**Supplementary Figure 21. Performances of gas-diffusion-layer-based electrocatalysis system powered by a Si solar cell. a** Photovoltaic and electrocatalytic current-voltage curves of the Si solar cell (black curve) and two-electrode $O_2$ evolution (red and blue curve). The intersections indicated the maximum PCE for the designed light reaction in each case. **b** Summary of current density (right *y*-axis), CO faradaic efficiency, electric PCE, and solar-to-CO PCE (left *y*-axis) in GDL-based systems with different electrolytes.

**Supplementary Table 1.** Calculations of the faradaic efficiency (FE), the electric power conversion efficiency (PCE), and the solar-to-CO efficiency.

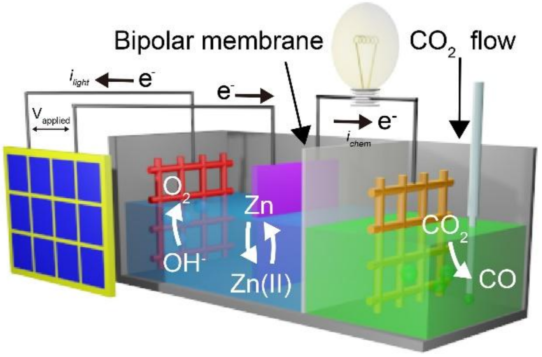


To gain the CO faradaic efficiency, the electric PCE, and the solar-to-CO efficiency, we recorded the applied voltage (labeled as $V_{applied}$), light reaction current ($i_{light}$), and carbon fixation current ($i_{chem}$) during the operation. The values of the three parameters in different cases are shown in the following table.

| Electrode | $j_{chem}$ (mA cm$^{-2}$) | $V_{applied}$ (V) | $i_{light}$ (mA) | $i_{chem}$ (mA) |
|---|---|---|---|---|
| Carbon paper | 2 | 1.96 | 4.35 | 0.65 |
| | 5 | 1.96 | 4.35 | 1.62 |
| | 10 | 1.96 | 4.35 | 3.22 |
| | 13.5 | 1.96 | 4.35 | 4.35 |
| GDL | 80<br>(3 M $KHCO_3$/6 M KOH) | 2.25 | 140 | 80 |
| | 80<br>(3 M $KHCO_3$&3 M KCl/10 M KOH) | 2.25 | 140 | 80 |
| | 100<br>(3 M $KHCO_3$&3 M KCl/10 M KOH) | 2.25 | 140 | 100 |

## Supplementary References


1 Mainar, A. R. *et al*. Alkaline aqueous electrolytes for secondary zinc-air batteries: an overview. *Int. J. Energy Res.* **40,** 1032-1049 (2016).

2 Cai, M., & Park, S. M. Spectroelectrochemical studies on dissolution and passivation of zinc electrodes in alkaline solutions. *J. Electrochem. Soc.* **143,** 2125-2130 (1996).
